# Public Safety Personnel Family Resilience: A Narrative Review

**DOI:** 10.3390/ijerph19095224

**Published:** 2022-04-25

**Authors:** Marilyn Cox, Deborah Norris, Heidi Cramm, Rachel Richmond, Gregory S. Anderson

**Affiliations:** 1 Department of Family Studies & Gerontology, Mount Saint Vincent University, Halifax, NS B3M 2J6, Canada; marilyn.cox4@msvu.ca (M.C.); deborah.norris@msvu.ca (D.N.); 2 School of Rehabilitation Therapy, Queen’s University, Kingston, ON K7L 3N6, Canada; heidi.cramm@queensu.ca (H.C.); rachel.richmond@queensu.ca (R.R.); 3 Faculty of Science, Thompson Rivers University, Kamloops, BC V2C 0C8, Canada

**Keywords:** family resiliency, public safety personnel, nonstandard work, work-family conflict, family time, instrumental support, emotional support, social support, family capabilities, trauma exposure

## Abstract

The families of public safety personnel (PSP) face demands that are unique to these occupations. Nonstandard work, trauma exposure, and dangerous work environments affect both workers and the families who support them. This narrative review aims to identify the stressors that PSP families experience and the support and resources needed to enhance family resilience. Due to a lack of research on PSP families, this review is a necessary first step to summarizing and interpreting a diverse body of research. The studies included addressed structural and emotional work-family conflict with reference to PSP sectors. A framework from the military family resiliency literature interprets the findings. Factors influencing family functioning and the availability and accessibility of resources provide clues about the type of skills and supports that PSP families rely on. Meaning-making, collaboration, a sense of coherence, and communication were identified as themes associated with intrafamilial processes. Extrafamilial themes included public perceptions, a lack of recognition for the roles families fulfill, and the need for information and education. The results suggest that the vulnerability of PSP families is variable and extrafamilial resources in the form of formal and informal supports are necessary to enhance family resiliency.

## 1. Introduction

There is an expectation that Public Safety Personnel (PSPs), such as firefighters, police, communicators, corrections, paramedics [1], make a commitment to protect the communities that they serve, which may require sacrifices and endanger their lives; fortunately, there has been increasing interest in the demands on and consequences for PSPs (e.g., [2,3,4,5]). However, the occupational risks and requirements that PSPs take on to keep our communities safe do not end with them but transfer through to their families [6,7,8,9,10,11]. Unfortunately, existing research about PSP families often focuses solely on PSPs and their careers, neglecting the effects that PSP careers have on other family members (e.g., [12,13,14]). It has been noted by many researchers that there is little evidence regarding the sacrifices and demands experienced by PSP families [6,7,8,9,10,12]. This narrative review aimed to synthesize and explore existing research to identify what is unique about PSP families and develop a more comprehensive understanding of the relationship between work demands and family processes. It has also served to identify significant gaps in the literature where research is needed. 

The existing body of literature disproportionally represents certain public safety sectors (e.g., police, firefighters) and is limited in scope. Many of the studies incorporated in this review have laid the necessary groundwork by identifying stressors (e.g., trauma, shiftwork) and outcomes (mental health issues) for individuals, but less is known about how these factors unfold and affect relationships between members of PSP families. It is the quality of relationships that is a determining factor for family resilience [15]. The concept of *family resilience* is used in this study to describe a dynamic process that enables families to cope with adversity [16]. Identifying and interpreting the nature of stressors and the family processes that are protective can inform resiliency intervention strategies for PSP families. 

There are similarities between the experiences of PSP and military personnel and by extension, a more developed body of literature on military family resilience can aid research on PSP families. Public safety (PS) organizations are paramilitary in structure, meaning they often utilize components of the organizational hierarchical reporting structure, tactics, and training methods of the military, as well as incorporating the often highly masculine and stoic subculture of the military [1,13,17]. Just as there are similarities in occupational risk across military and public safety sectors, so too are their shared experiences for their families. Military families face specific lifestyle dimensions and challenges such as relocation, separation, and risks of injury and death [18]. While certain PS sectors are impacted by relocation orders (e.g., RCMP [19]), this factor is more pervasive among military families. Many PSP families are also affected by separation, although it is experienced differently than the separation associated with military deployment. PSP can be away from home for days or weeks (e.g., wildland firefighters [20]), but the absence felt by families is more often related to long shifts and mandatory overtime [10,21,22]. PS work can also be dangerous and there are inherent risks to both physical and mental health for PSPs due to the nature and unpredictability of their work [1,23]. 

The parallels between military personnel and PSP serve as a basis for applying frameworks from the military families’ literature to PSP family research. The Cramm et al. [16] Synthesis of Military Family Resilience Factors, a pioneering effort to address the complexity of military family life, has been adapted and used in this study. This synthesis was applied to frame the relationship between family processes, intrafamilial factors, and ecological factors within the context of PSP family life. Combined contextual factors for PSP families such as rotating shiftwork, trauma exposure, mandatory overtime, and risks of injury or death challenge family resilience and set PSP families apart from the general population. The chronic and cumulative nature of stressors related to these contextual factors linked with the life stages of families (e.g., early career and young children) put PSP families at variable levels of risk and can result in a “pile-up” that depletes family resources [24,25]. 

Family belief systems, communication, and organizational patterns have been identified as family processes that influence family resilience by enhancing or diminishing capabilities [15]. A shared narrative, open emotional expression, and a flexible family structure with well-defined boundaries are family processes identified as protective in the military literature [26,27], which resonate in this study. Additionally, evidence of the impact of PS organizations, communities, policies, cultural norms, and public perceptions demonstrates how ecological factors permeate PSP family life, emphasizing the relevance of the adopted framework. 

## 2. Materials and Methods

A narrative review is designed to “summarize, explain, and interpret evidence” [28] (p. S1:11) of diverse subject matter and incorporate a variety of research methods. Due to the paucity of research that directly addresses the experiences of PSP families as a unique population with challenges distinct from the individual PSP member, a narrative review was deemed an appropriate choice for this study [29,30]. Narrative reviews are advantageous when research findings are dispersed across many disciplines, leading to a lack of consensus on gaps and priorities. Useful in the formative stages of research and theory development, normative reviews do not necessarily follow the prescribed protocols entailed in systematic and scoping reviews [31].

The purpose of this study was to identify what is unique about PSP families and understand the relationship between work demands and family processes. The literature that exists is primarily sector-specific (e.g., police, firefighters) and focused on specific challenges (e.g., PTSD, shiftwork). Thematic analysis facilitated the interpretation, merging, and reframing of evidence to generate new insights. Braun and Clarke [32] and Clarke and Braun [33] describe thematic analysis as iterative in nature, involving the comparison of emergent themes while still “collecting” data—or, as in this paper, while reading and reviewing the studies emerging from the narrative review. This allows the examination of possible connections between themes and how they vary. 

### 2.1. Inclusion Criteria

**Figure 1:**
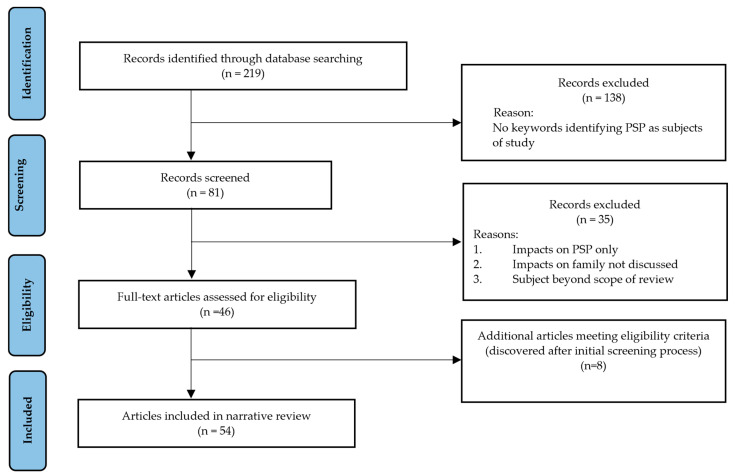
Diagram of narrative review search and article selection (adapted from PRISMA 2020 guidelines for systematic reviews) [34].

Qualitative, quantitative, and mixed methods studies, literature reviews, book chapters, and thesis dissertations were included. The following databases were searched: APA PsycNET, DOAJ, EBSCOhost, ERIC, Google Scholar, IBSS, JSTOR, MAG Online Library, ProQuest Dissertations, PubMed, SAGE, Scopus, SpringerLink, Taylor & Francis Online, and Wiley Online Library. Initially, databases were searched for keywords associated with structural work-family conflict (e.g., nonstandard work) and emotional work-family conflict (e.g., stress, trauma exposure) (*n =* 219). Keywords, eligibility criteria, and selection process were determined by the research team in consultation with a reference librarian. These articles were imported into MAXQDA software for further analysis. MAXQDA was selected due to the functionality associated with importing and coding pdf articles and the ability to do lexical searches, which allowed for the initial keyword searches prior to coding. The results were scanned for keyword references to PSPs, specifically, paramedics, ambulance services, firefighters, fire service workers, fire and rescue services, police, law enforcement professionals, law enforcement officers, RCMP, correctional staff, correctional officers, first responders, emergency service volunteers, public safety providers, public safety personnel, public safety employees, emergency medical service, emergency medical technicians, emergency service first responders, dispatchers, and 911 telecommunicators (*n =* 81). The abstracts from these results were screened to determine if the impact of the work on family members (spouses/partners or children) was the primary objective or an aspect of the research. Articles were excluded if the focus was on the work-family relationship insofar as it supported worker well-being, productivity, or retention rather than the health of the family as an outcome. Articles that focused on the worker were included if specific reference was made to the effect that the work had on family members (e.g., parenting, couple relationships). Reference lists from the articles included were checked for additional studies. One paper was excluded because it focused on emergency preparedness for PSP families, which was beyond the scope of this study. The included articles were vetted by the research team. Eight papers that met all criteria were added following reference list review and article screening. Figure 1 shows the process of the narrative review search.

### 2.2. Process of Analysis

Thematic analysis was aided by MAXQDA software. The selected articles (*n =* 54) were grouped by the type of demand in broad categories related to structural (e.g., nonstandard work schedules) and emotional interference (e.g., trauma) with subsets identifying family relationships and target populations. The output was exported to Excel and the major themes were mapped and shared with collaborators for input. Prominent themes were framed for discussion using an adaptation of the Synthesis of Military Family Resilience Factors [16]. 

## 3. Results

Literature that was included in the present review (*n* = 54) is outlined below:

- Target Population: police (*n* = 28); firefighters (*n* = 8); paramedics (*n* = 4); correctional officers (*n* = 1); dispatchers (*n* = 1); first responders (general) (*n* = 12)
- Family Relationship: couples (*n* = 16); spouses/partners (*n* = 12); children (*n* = 6); families (*n* = 13); and PSP (*n* = 7)
- Methodology: qualitative (*n* = 21); quantitative (*n* = 19); mixed methods (*n* = 7); and descriptive studies or systematic reviews (*n* = 7)
- Publication: peer-reviewed, available in English, published between 2000 and 2021 (*n* = 42); landmark studies published prior to 2000 (*n* = 4); thesis dissertations (*n* = 6); and book chapters (*n* = 2)
- Country: United States (*n* = 34); Canada (*n* = 11); Australia (*n* = 4); United Kingdom (*n* = 4); and New Zealand (*n* = 1)
- Year of Publication: 2010–2021 (*n* = 34); 2000–2009 (*n* = 16); 1980–2000 (*n* = 4) (see Table 1)

### 3.1. Adaptation of Military Family Resilience Synthesis

**Figure 2:**
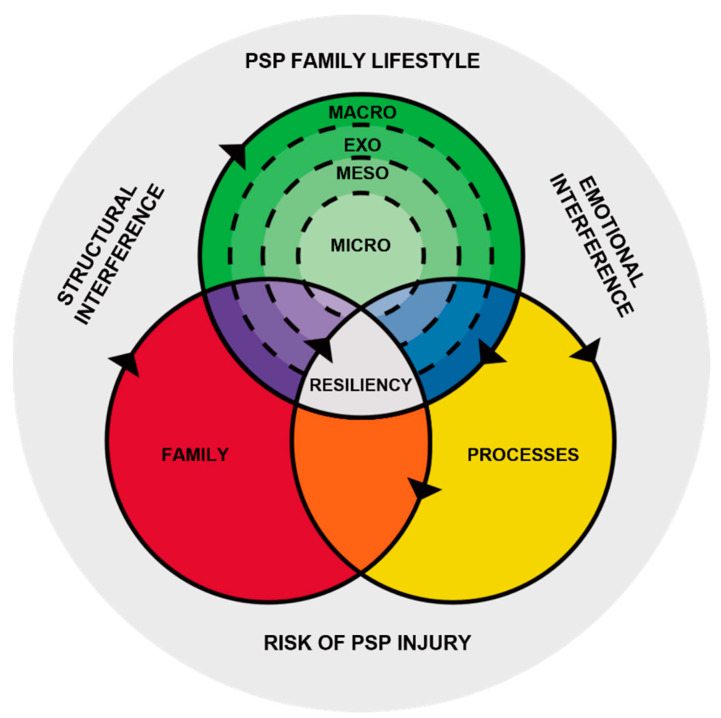
Synthesis of Public Safety Employee Family Resiliency Factors. Adapted from: Synthesis of Military Family Resilience Factors [16].

To capture the variability of PSP roles and the experiences of PSP families, the contextual factors noted in the Cramm et al. [16] Synthesis of Military Family Resilience Factors (relocation, separation) have been expanded and conceptualized as structural and emotional interference [59] (see the adaptation in Figure 2). Structural interference relates to rotating shifts, long hours, absences and separations, and relocations, while emotional interference relates to the stress and tension that arises due to the nature of the work (e.g., workload, trauma exposure).

Jackson and Maslach’s [59] analysis of data from police spouses found that quality of family life was related to emotional interference, but structural interference had a lesser impact. In other words, the quality of engagement with the PSP was more important than the amount of time that the PSP spent with family. As noted later by Jackson et al. [79], this brings about a causality dilemma given that long hours and shift work restrict family time, which could influence the quality of the available time. This has been illustrated by PSP family members who report that shift work schedules reduce opportunities for get-togethers on weekends, holidays, and special occasions with friends and extended family, limiting access to social support [6,36,37,38,39,40,41,42,43,44,45,46,47,48,49,50,51,52,53,54,55,56,57,58,59,60,61,62,63,64,65,66,67,68,69,70,71,72,73]. These results suggest that families can accommodate shiftwork, but time-based work-family conflicts, along with long and unpredictable hours, exacerbate the emotional toll that the job places on the PSP family. The findings are summarized in Table 2.

### 3.2. Structural Interference 

#### 3.2.1. Role Overload

The extent to which nonstandard work schedules are intertwined with the number, ages, and special needs of children, the availability of childcare, single parenting, and dual-career couples generates unique challenges for PSP families. A common sentiment shared by participants in qualitative studies was the feeling of being a *single parent* in a two-parent family when one of the parents was a PSP [6,7,10,47,57,69,74,77]. This is due to rotating shifts, unscheduled overtime, and unpredictable call-ins that place an unequal share of responsibility for childcare and managing the household on the non-PSP spouse. PSP family members often require recovery time due to long hours and shift changes which further reduces their availability at home. As Hill et al. [22] note, “the needs of the FRS [fire and rescue services] permeate the lives of relatives by the FRS being the clear priority of the family” (p. 398). The onus is on non-PSP spouses to be flexible and adjust to the scheduling demands of PSPs, which can lead to role overload, exhaustion, and resentment [11,48,51,57,67,72,74,77]. 

There is some evidence that the family roles of spouses are more egalitarian when the PSP is female. According to a Canadian study, female police officers tend to share parenting equally with their spouses, whereas male police officers spend significantly less time caring for dependents than their spouses who also work full-time outside of the home (22 versus 34 h) [56]. Fathers whose shift work allows them to be at home in the daytime often take on more childcare responsibilities than fathers who work standard hours [10,61]. Firefighters and their spouses in Sommerfeld et al. [73] considered shift work to be advantageous for childcare, though this can only have utility when the work hours of parents do not overlap and, therefore, is of limited advantage for those with rotating shifts. When workable, tag-team parenting was deemed beneficial for childcare but came at the expense of “couple time” [10,74]. Rotations of four days on and four days off also had advantages for family time; availability during the off shift provided relief for the non-PSP spouse [74]. Roth and Moore [72] found that families of emergency medical services personnel coped with tensions prompted by nonstandard schedules by negotiating role responsibilities and being flexible; however, participants pointed out that the division of labor in the home was not always equitable.

Childcare can present logistical challenges for single parents and dual-career families, particularly when both parents are PSPs or the non-PSP spouse also works nonstandard hours. Institutional childcare is often unavailable for nonstandard hours, and parents must rely on older children, extended family, or live-in nannies [10,35]. Some women in the studies postponed their own careers to care for their children because coordinating childcare was unworkable; others worked fewer hours or had to request time off to care for children because the PSP family member’s work with less flexibility and predictability was prioritized in spousal employment [10,48,69,74]. As Carrington [48] notes, “equity in household tasks might not be feasible in policing relationships, especially in the early phases of an officer’s career when rotational shiftwork and volunteer overtime are necessary” (p. 148). Spouses in the studies often expressed that they were committed to supporting PSP’s careers by managing the home and taking care of the family; however, this required both flexibility and sacrifices [6,7,10,22,48,57,69,72,74,77]. Young families must adapt simultaneously to the new demands of a PSP career, the couple relationship, and parenthood, making it difficult to define and balance family roles. 

Separation and relocation of PSPs are more variable than they are for military families but important to note since these aspects of the job have a significant impact on family roles and responsibilities. Wildland firefighters and disaster relief workers might be gone for days or weeks, for example, and transfers between detachments are common in police forces [41,48,56]. PSPs can also be required to be away from home for training or work extended hours with no notice due to critical incidents, putting additional demands on family members for childcare and household responsibilities [11,35,50,51]. Spouses of RCMP officers reflected on the challenges of relocation, noting that, while their partners moved to detachments with familiar roles and responsibilities, spouses had “to start their lives over and over again … [and] had to start from “scratch”” [48] (p. 69). Relocation compounds challenges for the careers of spouses who are unable to find work or childcare, particularly when they are posted to rural and remote communities and lose the instrumental support of extended family and close friends [37,48,74].

#### 3.2.2. Family Time and Socializing

PSPs must be available twenty-four hours a day, seven days per week (24/7) which requires rotations that include nights, weekends, holidays, and unscheduled call-ins. Despite the fact that families understand the 24/7 demands of PS work, the absence of the PSP on holidays was identified in empirical data as a significant source of stress for spouses of police officers [6,7] and echoed in qualitative studies for a variety of PSP occupations [9,10,38,42,47,57,69,72,73,74]. Some families developed strategies and celebrated holidays at different times [9,10,42], but this was not possible for events external to the family that are planned to accommodate standard work schedules such as weekend weddings, graduation ceremonies, and children’s sports [72]; moreover, young children did not always understand why their families did things differently [10]. Carrington [48] found that a few PSP families coped with the inevitable scheduling conflicts by adopting a “Family First philosophy”, which respected the time-based demands of PS work but nonetheless communicated that the family was their top priority; “they always knew or “felt” that they came first” (p. 120). In this way, the PSP schedule was accommodated with holidays and family events marked in unconventional ways rather than neglected. 

A primary issue for children of PSPs is that they spend too little time with the PSP parent [9,11,35,67]. Spouses and children reported that the PSP parents frequently miss children’s sports activities, school events, and special days [9,42,72]. Regehr et al. [10] and Watkins et al. [77] identified a tendency for firefighters to work a second job during off shifts, which could further reduce opportunities for family time. On the other hand, PSPs who had off shifts that allowed them to be home when their partner was at work were reported to spend more time in the parenting role [10,47,48,61,74]. There is uncertainty about the PSP’s physical presence in the family due to unscheduled overtime and call-ins; the chronic absence of PSP parents has been expressed in terms of anger and sadness for children [9,10,11,35,47,57]. There is evidence of PS organizations addressing this by allowing flexibility so that PSP parents can manage their schedules to attend family events and children’s activities by trading with co-workers or using *lieu days* (time off in exchange for working public holidays) [10,47,73]. However, there was also reference to the inflexibility of PS organizations [11,12,14,35,72], and study participants contend that schedule flexibility is largely associated with seniority, which disadvantages younger PSPs who often have greater childcare demands [14,48,73,74].

Factors such as the stage of the family, the number and age of children, and the rank of the PSP make the demands on each PSP family unique. For example, a sample of correctional officers under the age of 40 who were married with young children reported more time-based conflict with work and family than officers over the age of 40 with older children [14]. PSPs and spouses reported that their relationships were strained, and time alone as a couple was sacrificed to accommodate childcare and fulfill the demands in the early stages of the PSP’s career when there was less flexibility regarding shifts, transfers, and overtime [10,35,45,48,55,73]. Spouses of PSPs who were more advanced in their careers reported less disruption in part because the PSP had more flexibility regarding shifts and because the family had adjusted to the scheduling demands [10,14,74]. Many researchers have concluded there is a need for more organizational support and flexibility for PSP families early in their careers and those with young children to help them manage the structural demands associated with PS work [14,22,39,56,72].

Couple relationships can suffer and there can be resentment due to the time commitment required by the PS organization, time spent with coworkers off shift, or a lack of communication and intimacy due to fatigue and too little time alone as a couple [42,45,47,67,69,70,77]. Competition was a major theme in Bochantin [42], with PSPs and their spouses framing themselves as adversaries. Bochantin [42] concluded that, for the most part, “there appear to be no winners or survivors … between work and family especially pertaining to scheduling … because PSPs, as well as their families, have assumed a victim orientation rather than a survivor orientation” (p. 233). In contrast, other couples have espoused effective coping strategies to maintain their relationships by making time alone as a couple a priority [8,45,57,74] and maintaining contact during shifts (e.g., sharing a mid-shift meal together or a phone call) [48]. Regehr et al. [10] found that spouses who focused primarily on the benefits of nonstandard schedules and developed flexible strategies to manage both “couple time” and family time reported less stress. 

Difficulties maintaining social relationships outside of the immediate family and feelings of isolation and loneliness were also challenges associated with PSP schedules [10,37,57,69,73,77]. The greatest stress reported by spouses of police officers in an empirical study by Alexander and Walker [36] was a social life ruled by shifts, overtime, canceled leave, and transfers. Nonstandard schedules result in fewer opportunities for PSPs to socialize [14,60], and, in turn, spouses who take on a greater share of household and childcare responsibilities to accommodate PSP shifts have less time for outside relationships [72]. Some spouses do not attend parties or community events because they do not want to go alone [10]. Hill et al. [22] found that families did not want to live “separate lives” and chose to manage activities to include the PSP family member, who sometimes resulted in canceling plans or not making plans. In addition to scheduling challenges, families of RCMP and police may be transferred to new postings and lose the support of extended family and friends [6,48,74]. 

Spouses of firefighters in Regehr et al. [10] reported that they depend on extended family for both social and instrumental support and it was particularly difficult when these people did not live nearby. To prevent the isolation that can accompany the lifestyles of PSP families, spouses commented that it was important to be “self-reliant”, and make time for their own interests and maintain friendships [10,48,69,72,74]. The mutual support of other PSP families was also identified as helpful and allowed family members to share common experiences regarding the everyday demands associated with PSP schedules [7,10,37,47,48,73,74]. 

#### 3.2.3. Routines

The maintenance of routines and rituals is particularly important for pre-school children [80] and supports communication between parents and adolescents [81]. The degree to that routines are impacted by nonstandard work depends on the type of shift. Studies suggest that the evening shift (work time between 2 p.m. and midnight) is the most difficult for families because parents are not available for after-school sports, evening meals, homework, or bedtime rituals [72,80,82,83,84,85]. The need for daytime sleep by PSPs working the night shift can be problematic for families with toddlers who simultaneously try to support the PSP’s sleep recovery and interact with their children [11]. Parents in Regehr et al. [10] reported that their adolescent children had difficulty adjusting to changes in rules and routines that accompanied the PSP parent being on shift for four or five days than being at home for the next four or five days. Rotating shifts (days to evenings to nights) and call-ins are also challenging. Variable work time and unpredictability make it difficult to maintain family routines and plan activities [7,38,72]. As family members in Hill et al. [22] put it, families develop “an ever-changing “non-routine”” (p. 399). 

Night shifts and emergency calls during the night can interrupt sleep and be accompanied by worry for the PSP’s safety and a subsequent lack of sleep for family members, which results in tension and stress [21,75,77]. Spouses have reported feeling exhausted due to hypervigilance during night shifts, having their own sleep patterns affected by the PSP’s rotating shifts, and the challenge of supporting the PSP’s sleep recovery (e.g., keeping children quiet) [6,72,77]. PSPs who prioritize family responsibilities over sleep recovery, though well-intended, accumulate sleep loss which can impact families due to negative affect and poorer interactions with children and spouses (e.g., fall asleep or become agitated) [77].

Participants in Roth and Moore [72] expressed that nonstandard schedules disrupt the “home rhythm” by altering mealtime and bedtime routines, along with family activities. In contrast, the standard workday was reflected on by one family member as being “monotonous” [72] (p. 465), and, although nonstandard schedules were admittedly challenging, some participants felt they provided more flexibility for daily activities [10,47,48,69,73,77]. 

#### 3.2.4. Ambivalence 

Family members appreciate the essential roles that PSPs fulfill, and the importance of this work influences how families function and perceive their roles. Many family members express a sense of pride in the work of PSPs and their roles in supporting the PSP, but this is tempered by ambivalence [7,8,36,42,74]. Family interactions are often controlled by nonstandard schedules that require spouses and children to make significant sacrifices to support PSPs’ work commitments [6,10,22,48,72,74]. In some cases, women forgo opportunities for paid employment and accept traditional gender roles in the home to accommodate PSP schedules or participate in full-time paid employment but remain the primary caregiver for children [6,10,47,67,69]. Others develop strategies and negotiate and share responsibilities for both childcare and the household with PSP partners (e.g., tag-team parenting) [35,47,65,72]. Duxbury et al. [56] note that male police officers report that they are taking on a more egalitarian role in families, which is exerting pressure on organizations to adjust outdated expectations regarding traditional family roles. Family members believe that the instrumental support they provide so that PSPs can fulfill work commitments is substantial but largely goes unnoticed by PS organizations [22,37,48,76].

### 3.3. Emotional Interference

#### 3.3.1. Behavior-Based Conflict

Certain types of behaviors that are necessary for PSPs to perform work roles become problematic when they spill over into family relationships [86]. Miller [67] notes that some police officers can compartmentalize *work* and *family*, but others find it difficult to “turn off the job”, particularly when they have worked long hours and have been exposed to trauma [60]. Participants in Bochantin [42] practiced the “intentional separation of work and family roles” (p. 229), which was deemed necessary by the PSP parents in the study but also resulted in the children “withhold[ing] their “true” feelings” (p. 230) because they were afraid this would distract the PSP parent from their work.

PSPs exposed to accidents and criminal activity are at risk of transferring protective, suspicious, hypervigilant, and authoritarian behaviors that are important to their roles in the workplace into the home environment [67]. Johnson et al. [60] researched authoritarian spillover and found that forty percent of police officers in their sample reported that they had been abusive to spouses during a period of six months prior to the survey. Family members did not report physical abuse in the qualitative studies included in this review; however, there is evidence that after a long or difficult shift, PSP families can be subjected to anger and irritability from PSPs [10,36,45,46,47,57,59,63,69,74].

Children describe some PSP parents as overprotective [11,21] and spouses have described PSP partners as controlling and protective of both themselves and their children, which creates tension for families [7,8,67]. Agocs et al. [35] describe how police mothers “engage in danger-protection parenting practices to prevent their children from becoming victims or offenders” (p. 267), emphasizing how police work influences parenting perceptions and behaviors. Children in Helfers et al., [21] reported that police parents made them feel safe but restricted freedoms, such as denying them access to social media. This corresponds with older children in Bochantin [42] who felt that they were “interrogated” by the PSP parent.

#### 3.3.2. Ambiguous Loss

In addition to the uncertain physical absence of PSPs due to unscheduled overtime and call-ins, the psychological absence of PSPs who are present but withdrawn from their families because they are either physically exhausted or processing a traumatic event manifests as an ambiguous loss. Boss [24] posits that ambiguous loss, the uncertainty of physical or psychological presence, poses the greatest challenge for family functioning and was a common theme in qualitative studies [9,10,47,57]. As the wife of a first responder reflected on the breakdown of communication with her husband, “[i]t’s like he’s here, but he’s not” [9] (p. 48). The PSP is present in the family but unable to provide instrumental or emotional support, which “creates significant unpredictability regarding the boundary that surrounds the family, its rules, roles, and patterns of relating” [10] (p. 425). The ambiguous loss was more pronounced for family members when PSPs suffered from PTSD [41,61,76].

Family members reported anticipating withdrawal by “reading and monitoring” the PSP [10,22] and giving the PSP “space” [72] and “downtime” [42] when they returned home to allow recovery time. When spouses were also PSPs or in an allied occupation like healthcare, PSPs were more likely to communicate with their spouses about their work at the end of a shift which allowed them to “decompress” and aided the transition from work to family [45,48,57,67,69,72].

#### 3.3.3. Crossover

Whereas spillover refers more generally to the effects of the work environment on the home environment, the crossover is a subset described in the work-family conflict literature as “interindividual transmission of stress or strain” [87] (p. 901). In other words, the stress that the PSP experiences in the workplace *crosses over* or is transferred to family members. Spouses described being a “sounding board” for first responders [9] (p. 48) and some felt discomfort and anxiety with the details that PSPs shared [57,69,74]. In a mixed-methods study, Friese [6] found that spouses of law enforcement officers who experienced high levels of job-related stress experienced equal or greater levels of stress arising from their relationships. Similarly, Roberts and Levenson [70] reported that negative affect on days with a high level of work stress for police officers corresponded with low positive affect for their spouses. Spouses of law enforcement professionals in Landers et al. [8] “described experiences with nausea, intrusive thoughts, anxiety, shaking, confusion, mood changes, fear, and worry stemming from their own responses to the LEP’s [law enforcement professional] exposure to traumatic events” (p. 314). 

Children are also vulnerable to crossover effects. In a study associated with the World Trade Center (WTC) attacks, Hoven et al. [58] posit that children of first responders exposed to dangerous work environments are at greater risk of mental health problems than the general population, in part, due to fear, worry, and impaired parenting. Kishon et al. [64] found a higher incidence of separation anxiety disorder among children whose fathers were first responders with reports of “recurrent excessive distress when anticipating or experiencing separation from parents, reluctance/refusal to go to sleep without being near the parents, and physical symptoms (e.g., headaches, stomach aches, nausea) when separation occurs or is anticipated” (p. 911). Helfers et al. [21] concluded that the stress of police work could transfer to children, affecting behaviors and academic achievement. All the children in this study expressed fears about the safety of their police parents and just under half of the participants said that the police parent was reluctant to talk about their work which may have exacerbated worry and fear about the dangers. Helfers et al. stressed the importance of mental health initiatives for children at an organizational level. 

An aspect of crossover that is getting increasing attention is secondary trauma, which arises from an “empathetic relationship with the traumatized individual” [66] (p. 1) and results in a non-exposed individual (e.g., a family member) developing posttraumatic stress symptoms (PTSS). Using a secondary trauma stress scale, Alrutz et al. [37] found that twenty percent of their sample of spouses of emergency responders (*n* = 646) were at moderate to severe risk of secondary trauma, which corresponds to Davidson et al. [52] who concluded: “that the presence of PTSD in trauma survivors [police officers] fosters psychological disturbance in their intimate partners” (p. 46). Following the WTC attacks, Duarte et al. [53] found “probable PTSD” among the children of emergency medical technicians, police officers, and firefighters. The importance and need for education and information regarding the prevention and early treatment of secondary trauma for PSP families are prominent in the literature [8,37,66,76].

Spouses who were also PSPs or in allied health occupations said that they could understand and cope with the information that the PSPs shared regarding traumatic events [48,57,67,69,72]. In other cases, PSPs were reluctant to discuss the “gruesome” details of their work at home to protect family members from crossover effects [45,47,61,75]. To illustrate the problems associated with PSPs relating to ambiguous details or non-disclosure, Bochantin [43] introduced the concept of “sensetaking” to describe how family members can erroneously reconcile missing information, potentially resulting in even greater worry or anxiety. Researchers conclude that strategies for open communication between family members that are age-appropriate [54], culturally appropriate [45], and constructive favor better outcomes versus avoidance behaviors or misthought attempts by PSPs to protect family members [44,71,72].

Opportunities for PSPs to debrief with peers after a traumatic event were considered advantageous by some family members to help manage the after-effects of trauma exposure and reduce the risk of crossover at home [10,74]. However, formal debriefing is reserved for major events and offered at the discretion of the organization [39,73]. Informal debriefing was available for some PSPs but not available for family members, and when social support is garnered from co-workers after a shift, it conflicts with family time [10,69,73]. Recommendations by both study participants and researchers highlight a need for organizations to include family members in debriefing when there is a significant event to support the PSP family member and not become traumatized themselves [6,36,37,48,53,74]. Support from organizations in terms of “induction events” was also recommended to familiarize family members with the PSP’s work environment, the risks, the impact on both PSPs and families, and available resources [37,48,51].

#### 3.3.4. Identity

In a 24/7 economy, shiftwork is common in the general population, but the essential nature of PS work, the trauma exposure, and the risks of injury or death combined intensify work demands for both the PSP and their family. The work identity of the PSP and their commitment to the PS organization spills over to the family due to the unavoidable structural and emotional demands of the work. Family members share the PSP identity by describing themselves as a “cop’s kid” or “cop’s wife” [48,67], or “half the badge” [48] and many families adopt the “first responder lifestyle” [9] by choice, obligation, or necessity [11,22,47,72,74]. Families become immersed in the PSP *way of life* due to expectations of loyalty to the organization [48,67], the importance of PS work [11,22,57,74], and work schedules that require families to “expect the unexpected” [22] (p. 398). 

In some cases, family members share PSP responsibilities such as answering fire calls for volunteer firefighters [50] and being on “the front line” with RCMP partners in remote detachments [48] (p. 70). Studies also indicate that family members are pressured to adjust their social behaviors to meet the expectations of the PS organization and the public (i.e., responsibility, discretion, confidentiality) [45,48,60,67,74]. Miller [67] notes that “adolescents are often caught between feelings of loyalty and pride in their parent’s work and anxieties about peer rejection because of common pejorative attitudes toward authority figures such as police officers” (p. 29). Children in Helfers et al. [21] reported being harassed and bullied by peers because their parents were police officers. 

The public both depends on PSPs and holds them accountable for public safety as a collective, yet public perceptions of PSPs are variable based on the work that they do. Study participants shared that “the public put firefighters on pedestals” [10] (p. 428) which corresponded with the “hero” status noted in Bochantin [11], Carrico [47], and Sommerfeld et al. [73]. Studies associated with the WTC attacks also suggest that positive appraisals by children regarding the PSP parent’s occupation (i.e., ‘hero’ status of firefighters) may be a protective factor that enhances resilience [53,58]. In contrast, police have been regarded with “public suspicion and disdain” [78] (p. 98), a negative public opinion that persists in very recent literature on police and their families [21,38]. Over half of the children in Helfers et al. [21] described being bullied by peers and “89% reported that people directed unfair comments toward the police” (p. 246). There was also a perception of a “lack of respect” for paramedics in Regehr [69] (p. 102). Public opinion regarding other types of PS work (e.g., correctional officers, 911 dispatchers) is unclear, possibly due to less presence in mainstream media and a gap in the literature. 

Despite these challenges, a sense of pride was prevalent in the literature, with families strongly identifying with the PSP family member and the PS organization [7,8,9,10,22,36,45,48,50,53,67,69,73]. Family members in Hill et al. [22] demonstrated knowledge of organizational policies and procedures, emphasizing that the PSP’s role as a shared investment and commitment. This strong identification with the organization sometimes develops into a sense of belonging and a network of support for PSP families [7,22,48,74]. In other cases, family members felt that both formal and informal support for family members was lacking [10,37,57]. 

There is an emphasis in the literature regarding the perception of a lack of recognition for the roles that family members fulfill to support PSPs and, by extension, PS organizations [22,37,47,48,74,76]. Waddell et al. [76] describe this phenomenon as a “sense of invisibility”, resulting in the absence of services and support for family members. The paucity of research on the impacts of PSP work on family members has been widely acknowledged, further indicating that this group has largely gone unnoticed [7,9,35,45,76].

### 3.4. Risk of Injury or Death

PSPs responding to dangerous situations are vulnerable to physical injuries and operational stress injuries, which can be life-threatening and the stress from this working environment can spill over into family life [8,12]. One aspect of this is the negative effect that can accompany high levels of job stress [70] and result in emotional reactivity or withdrawal of the PSP in the family environment [10,62]. The challenges for family members subjected to the PSPs’ stress responses are significant (ambiguous loss, crossover), which was addressed previously in the context of emotional interference. A significant stressor that accompanies spillover is the stress and tension experienced directly by family members due to uncertainty about PSPs’ safety at work. Although an earlier study by Alexander and Walker [36] suggested that dangers related to police work do not significantly impact families, more recent studies indicate that dangers are a primary stressor for PSP family members [8,9,10,42,43,45,47,48,64,74,75].

#### 3.4.1. Life-Threatening Work

Some spouses expressed the constant fear of a “knock” on the door [22,75], while others said that they tried not to think about it [22]. One study participant shared that the fear was amplified when the death of a PSP was reported in the news [8]. Fear was also heightened for family members when PSPs worked unscheduled overtime because the PSP family member could not contact them, and the organization did not inform them [37,73]. Helfers et al. [21] point out the probability of police being injured is magnified through mainstream and social media and the negative accounts of police officers have drawn attention which has increased children’s worry for their parents’ safety. 

Strategies to deflect the fear and worry associated with the inherent dangers of PSP work are prevalent in the literature. Bochantin [43] explored sensemaking by PSP families who use humor to ease the tension, which was helpful but ineffective in addressing the real dangers of the PSP job. The reporting of critical incidents through social media was a theme in Friese [6], with spouses of police officers both experiencing fear upon hearing news and relief as more information was quickly made available. Parents expressed concern that children worried too much about their safety because they got their information from the news and television shows, but they also wanted to protect them and were unsure what information they should provide [43,47]. In turn, children who have concerns about the PSP parent’s safety are sometimes reluctant to talk to their parents: “What good is it for my dad to worry that I’m worrying about him?” [43] (p. 287). Children who could talk to their parents about their work and the risks expressed fewer concerns about their safety [47].

Some of the more successful strategies to manage fear and worry about the dangers included open communication regarding the risks with couples making specific plans [47,74]. Family members who had an opportunity to connect with PSPs during a shift were reassured about their safety [48]. It was also noted that the intensity of the fear could also dissipate over time; one woman described constant worry and nightmares when she was first married to a firefighter, which she no longer experiences [10]. Family members who had confidence in the PSP’s skills and training and organizational procedures assessed the probability of injury or death to be low [10,22,47,48].

#### 3.4.2. Injuries

A further concern is an impact that physical or operational stress injuries (OSIs) can have on the family. There are a number of chronic diseases associated with shiftwork and work exposure (e.g., toxic fumes, HIV) as well as acute and disabling physical injuries associated with unpredictable and dangerous work environments [77]. OSIs are also more common among PSPs than in other occupations due to repeated exposures to danger and trauma [76]. Although there is a lack of research on the effects of OSIs on PSP family members [76], a scoping review by Norris et al. [88] in the military literature provides evidence of the negative impacts of OSIs with emphasis on secondary trauma. Along with the mental health risks, spouses often become primary caregivers for injured PSPs [8,9], which may result in role overload. There are also financial concerns, as the spouse of an RCMP officer noted: “Something could happen to him, he could be injured permanently, and I need to be able to support the family” [74] (p. 176). The families of injured PSPs may require significant adjustments with an increased demand for both instrumental and emotional support. 

## 4. Discussion

A review of the PSP family literature reveals the extent of demands placed on PSP families as well as the heterogeneity of PSP work which shows variation in the type of PS organization, the role of the PSP within the organization, and the nature of the critical incidents that arise. There will also be variability in PSP families based on the developmental stage of the family, the number and age of children, the availability of extended family and friends, and the health and wellbeing of family members. Nonetheless, adapting to structural and emotional interference and the risks of injury or death combined sets PSP families apart from the general population. The findings align with our adaptation of the Synthesis of Military Family Resilience Factors [16] in the identification of intrafamilial factors and family processes layered within the ecological model that foster family resilience.

### 4.1. Intrafamilial Factors

As Cramm et al. [16] note, “[e]nvironmental presses [chronic stressors] may elicit a steady response, whereas a series of environmental pulses [acute stressors] may disrupt equilibrium in the family system” (p. 628). The demands on young PSP families can be significant due to less control over work schedules in early careers and fears and worries regarding PSP safety which are compounded by the pressures associated with couple relationships and parenthood. However, as young couples are repeatedly exposed to the demands, many adjust to both family life and PS work. As Rutter [89] notes, stressors can have a “steeling effect”, strengthening and protecting the family through inoculation. Family resilience as a process focuses attention on family demands over time and across ecological systems for prevention and intervention [90].

Cascade modeling in the resilience literature shows that a build-up of risk factors can deplete resources and intensify vulnerabilities, whereas a build-up of protective factors can enhance capabilities and promote resilience [91]. The adaptation of the Cramm et al. [16] synthesis has utility in delineating and recognizing the interplay between the contextual factors of structural interference and emotional interference. Structural interference identified in this review included nonstandard schedules, dual-career parenting, an inequitable share of family responsibilities, caregiving due to injury or illness, separation, relocation, and prioritization of the PSP career. Emotional interference was primarily related to behavior-based conflict related to spillover of PSPs’ roles, critical incidents, and trauma exposure. Chronic stressors that arise for family members include role overload, disturbed sleep and hypervigilance, fatigue, disrupted routines and plans, ambiguous loss, restricted family time and social support, and acute stressors such as on-the-job injury or illness, family violence, and secondary trauma. The potency of these stressors is influenced by whether they serve as “hindrance stressors”, inhibiting healthy family functioning, or “challenge stressors”, enhancing family resilience by prompting families to make adjustments and develop strategies to cope with demands [92]. 

### 4.2. Family Processes

Patterson [93] defines capabilities as “resources, which are what the family has, and coping behaviors, which are what the family does” (p. 215). Capabilities are directed by “family processes”, an integral part of the Cramm et al. family resiliency synthesis [16]. Belief systems, organizational patterns, and communication are the components of family processes charted in Walsh’s [15] family resilience model that buffer stress and enable families to cope with adversity. Family stability depends on the consistency and quality of these resources to counter family demands [94]. The unique context of PS work (structural and emotional interference and risk) amplifies the interdependence of family members for support and continuity. The following discussion on family processes focuses on family strengths and resources noted in the findings, which support positive cascades.

#### 4.2.1. Belief Systems

Meaning-making has been identified as a key element in resilience models [93,95,96], highlighting the importance of shared narratives. The impact of demands can be attributed to situational meanings, which are the family’s subjective assessment of their circumstances and global meanings, which are associated with the family’s schema and shared identity [93]. Mancini et al. [97] found that military families who adopt a family schema that upholds the importance of the military mission and self-sacrifice more readily accept demands framed in family values. Applied to PSP families, the qualitative literature suggests that families who were knowledgeable about PS work (particularly those in allied occupations) and valued the PSP role more readily accepted the structural and emotional challenges. A sense of commitment and pride in the PSP role was prevalent, which tempered the reactions of family members to the chronic disruptions. For example, the firefighters involved with the WTC attacks were appraised positively by their children due to their “hero status” and researchers posit that this may have contributed to resilience and a lower rate of mental health problems for these children [53,58,64]. 

Some families incorporated a “Family First” philosophy [48] while honoring the time commitments to the PSP career by adjusting but not neglecting holidays and family activities. A firmly held belief that the family was a priority allowed families to reframe their experiences. Families who immersed themselves in the PSP *way of life* normalized their experiences with rotating schedules and unscheduled overtime and learned to “expect the unexpected” [22] (p. 398). Although Bochantin [42] concluded that families “assumed a victim orientation” (p. 233) regarding PSP schedules, there was also evidence that some families focused on the advantages of nonstandard work, such as daytime availability for parenting and family activities. The family’s subjective assessment of work interference can influence whether these chronic disruptions serve as hindrance stressors that inhibit or challenge stressors that facilitate family resilience.

Developing a sense of coherence is a hallmark of resilience [16] which was operationalized by Lavee and Olson [98] as “acceptance of stressful events and confidence in the family’s capabilities to handle difficulties” (p. 788). Families believed that their capabilities were sufficient to manage the challenges. The family’s capacity to cope with the inherent risks associated with PSP work depends largely on the family’s understanding of the PSP’s role and the real dangers. Those who trusted the skills of the PSP and the training and safety procedures of the PS organization indicated that they were less affected by these stressors. Similarly, those families who made plans should an injury, illness, or death occur also reported feelings of efficacy. A sense of coherence was spawned by the comprehensibility of the dangers making the risks manageable and challenging families rather than overwhelming them. 

Due to the intrusive nature of PSP work associated with structural and emotional demands, PSP families have more of an investment and connection to the PS organization than is typical in other occupations. Through their commitment and loyalty to the PS organization, some families make meaning by developing a “shared sense of belonging” [22] (p. 398) and adopt a shared identity and narrative. Evidence of this was conveyed by the children of police officers in Helfers et al. [21], who were bullied by peers and subjected to negative comments on social media; the authors noted that “the children interviewed believe people are ill-informed about the reported police incidents” (p. 251). The capacity of these children to counter the negative messages in the context of their family belief system is a measure of family resilience.

#### 4.2.2. Organizational Factors

Organizational factors described by Walsh [15] include “flexible structure, connectedness (cohesion), and social and economic resources” (p. 10). Challenged by the unpredictability of nonstandard schedules and trauma exposure, the need for adjustments and flexibility as described in the literature as a chronic stressor for PSP families. As noted previously, prioritizing the family encouraged families to restructure to spend quality time together. Similarly, couples, particularly dual-career parents, who had significant time demands, found creative ways to spend time alone. Spouses and PSPs pointed out the advantages of nonstandard schedules, which included more time for parenting for those who had full days of off shifts during the week and more egalitarian parenting when the PSP was a woman. It was evident in the qualitative studies that flexibility was imperative with PSP families adjusting to a new normal based on rotating shifts and unscheduled overtime: “an ever-changing “non-routine”” [22] (p. 399). 

Whereas Walsh [15] focused on the need for flexibility, Olson’s [96] Circumplex model distinguished between high and low measures of flexibility, with those at either end of the spectrum being dysfunctional and diminishing family resilience. Olson et al. [99] more recently cautioned that there is a need to renorm “if the family norms support these more extreme styles” (p. 203). Though it was clear that there is a threshold, particularly when childcare is involved, there was also an indication in the literature that PSP families can adjust to the structural demands of PSP work and adopt new norms. Aside from nonstandard schedules, the children of police officers [21,41] and police parents themselves [35] perceived that their families were subject to rigid rules due to overprotective parenting. On the one hand, these children felt safe, but they also felt they were subject to more restrictions and suspicion than their peers. Well-defined boundaries have been identified as facilitative for family resilience in the military literature; however, rigidity can be a risk factor for families [27]. 

Family cohesion focuses on reciprocity and collaboration [15]. Integration of the PSP in the family unit depends on both the quantity and quality of time available to develop bonds. Some families emphasized that they did not want to live separate lives from the PSP and accommodated the scheduling demands of the PSP so that they could spend time together as a family. Maintaining a connection with the PSP during a shift (phone call or sharing a meal) provided opportunities for communication and provided reassurance regarding the PSP’s safety. Though challenging, families also found ways to support sleep recovery and spouses accepted additional roles and responsibilities to support the family when the PSP was working long hours. PSPs who disengaged from family interactions or were emotionally reactive (i.e., irritable, angry) due to physical exhaustion or trauma exposure increased demands for families. Reading the signs and allowing the PSP recovery time after a shift were adjustments that family members made to help facilitate the PSP’s recovery after a stressful shift. In some cases, debriefing with a spouse who was knowledgeable of the PSP’s work role helped the PSP transition from work to family; however, this could also put the spouse at risk of negative affect or secondary trauma. The physical and psychological absences of PSPs put families at risk, while valuing family time and making family time a priority are protective and align with resilient families [91,100].

Social and economic resources are organizational factors that also influence family resilience. Meaningful relationships outside of the family, financial wellbeing, and sufficient community resources (e.g., health, education) help families build capacity. Social support is of primary importance because it provides family members with feedback and helps them adjust their expectations and their coping behaviors to enhance family resilience [93]. Isolation was a common theme in the literature due to unpredictable work hours, the type of PSP work, and relocations, particularly to rural and remote regions, which limit social interactions for families. Military children often feel isolated from their peers due to their unique circumstances, threatening their mental health; however, a supportive family environment has been shown to buffer against these risks [101]. Similarly, Helfers et al. [21] note that there is a perceived lack of peer support among children of police officers, and they depend primarily on family for support. They add that unlike military children who have opportunities to connect with peers in the larger military community, informal support of this nature is not generally available to children of police officers. Jackson and Maslach [59] stressed the need for informal support networks for families to share information and coping strategies to alleviate a sense of isolation. 

Issues related to economic resources were less prominent in the PSP literature, with mixed messages regarding financial security depending on the type of PS work and the regional location. The prioritization of the PSP career, however, made families more dependent on the PSP for family income, particularly when spouses postponed their careers or worked part-time to manage childcare. Financial pressures on single-earner families and an awareness that the inherent dangers in PS work could end the PSP career at any time are added stressors for families. The risk has been managed by some spouses by prioritizing and maintaining their own careers to ensure financial stability for the family. Economic hardship puts families at risk and is a significant factor for family resilience [15]. 

#### 4.2.3. Communication

Communication facilitates belief systems and organizational factors. Communication processes focus not only on the message but on how it is being conveyed. As Riggs and Riggs [102] state, it is the “quantity and quality of communication” that enhances or diminishes family resilience (p. 685). Reluctant communication was a common theme in this study, with PSP and non-PSP family members avoiding conversations about the trauma and dangers associated with PSP work. Spouses in allied occupations and those who were more knowledgeable about the PSP role could communicate more openly with the PSP while others experienced discomfort. 

Attempts by PSPs to compartmentalize work and family and protect family members from secondary trauma could interfere with open communication. The PSP who withholds information may unintentionally neglect the needs of family members to understand the PSP’s work role. Children, aware of the stressful nature of the PSP job, did not want to add to that stress by sharing their concerns with the PSP parent. In the distinction between sensemaking and “sensetaking”, Bochantin [43] demonstrated that humor could ease tension for PSP families and the appropriate use of humor is identified as a resource that enhances family resilience [15,93,103], but it can also create confusion. Family members who received no information or unclear messages about the PSP role had greater fears and worries than those who had open communication. Findings by Helfers et al. [21] and Bochantin [43] suggest that children who get most of their information about the PSP parent’s role from TV or social media tend to exaggerate the risks. Clear communication and information about the PSP role allow family members to interpret the dangers more accurately and avoid unnecessary worry, yet questions remain about age-appropriate disclosure and the risks of secondary trauma. 

Open emotional expression is an important aspect of communication that involves mutual empathy and respect for differences [26]. This is particularly challenging when a family member is processing trauma and may be avoidant or reactive. Informal and formal debriefing for the PSP during or after a difficult shift was valued by family members, but they also voiced the need to include family members in debriefing to maintain supportive communication and reduce the risk of secondary trauma. It was also evident that the prioritization of the PSP role and the importance of the work undermined the sacrifices that family members made and made it difficult for them to express feelings (e.g., sadness, worry, resentment). Withholding strong emotions creates tension and can impair family communication putting families at risk [27]. Saltzman et al. [27] add that a shared narrative and prioritizing quality interactions facilitate family resilience by nurturing family relationships.

Clear rules and boundaries and the maintenance of family routines support family resilience [91] but are difficult for PSP families to manage. Rotating shifts, unscheduled overtime, and call-ins can result in inconsistencies in family roles, parenting, and routines. PSP families are constantly making adjustments, negotiating roles, and problem-solving to maintain family stability. The capacity of the family to utilize family resources, resolve conflict, and develop viable solutions for their unique circumstances is a measure of family resilience [91]. The literature suggests that PSP families can adjust to structural interference over time by abandoning counterproductive practices and finding more constructive ways to cope, which prompts the demands to serve as challenge stressors and fosters family efficacy [92].

### 4.3. Extrafamilial Factors

In addition to intrafamilial factors, a shortage of institutional daycare for nonstandard hours, gendered expectations, and a lack of recognition for families by PS organizations and the public are extrafamilial factors that influence family functioning. PS organizations, communities, policies, cultural norms, and public perceptions permeate the family system as we shift from the micro level to the macro level of Bronfenbrenner’s [104] ecological systems model. As Cramm et al. [16] note, directing attention to those exchanges across ecological contexts that affect family meaning-making and resources can inform our understanding of those factors that inhibit or enhance family resilience. 

The importance of informal social support was identified in the literature. Both instrumental support (childcare) and emotional support from extended family and friends were resources that allowed PSP families to cope with both the structural and emotional demands. The lack of institutional daycare for nonstandard work requires that families find their own means of childcare, relying on informal support or absorbing the cost of live-in nannies. In a developmental context, young families with PSP parents early in their career often have the least flexibility in their schedules and the greatest family demands. Informal support is needed to balance work-family demands. When this support is not available, PSP families with children may have to make significant adjustments to maintain stability, such as postponing the career plans of the non-PSP parent, thus reducing the family’s financial resources.

Most spouses in the studies were female, with male partners in the PSP role. The prioritization of the PSP career and the time-based demands resulted in many of the spouses fulfilling traditional sex roles in the home, particularly in childcare. A recent study shows that female spouses of police officers who work full-time do significantly more work in the home than their partners [56]. Interestingly, couples are more egalitarian when PSPs are female. There is also an indication that the fathering role has increased importance for PSPs, a commitment that may require significant change at an organizational level to accommodate.

PS organizations primarily direct both formal and informal support to PSPs rather than families; however, the unique nature of PS work affects families and requires a commitment from the family as a unit. The findings suggest that recognition and organizational support for families lack, putting them at further risk. Being valued for your role is a protective factor that reinforces a sense of purpose [100]. Instrumental support in the form of education and information (e.g., induction events, family debriefing) and the facilitation of informal support networks of PSP families is critical for many PSP families. By supplementing intrafamilial resources, the provision of key resources by the PS organization can foster family resilience.

Public perceptions and expectations regarding PSPs and their families are also important to this discussion. The bullying of school children by police officers and the negative messages in both mainstream and social media are added stressors for these families. The highly-publicized nature of incidents in the media also amplifies the risks and dangers which affect both adults and children. In contrast, the “hero status” associated with the firefighter role is reflected in children’s positive appraisals of the parent’s work. Positive feedback regarding the PSP role develops or reinforces a sense of pride which enhances family resilience and becomes part of the families’ global schema. The absence of some types of PS work in both mainstream media and research potentially renders PSP families invisible. Due to the cumulative demands, spillover, and the risks to mental health, there is a need for cultural competence in communities and schools to appreciate the roles of PSP families and the unique challenges that they are confronted with.

### 4.4. Limitations

This narrative review was primarily exploratory and aimed at synthesizing existing research that identifies factors that influence the resilience of PSP families; therefore, the quality of these studies was not evaluated. There were discussions with collaborators and the support of a reference librarian in the selection of databases and keyword searches; nonetheless, keyword searches are not exhaustive, so it is possible that replicating the study would return different results. There is limited research on PSP families, and it is evident that research interest in PSP families is in its infancy, with almost half of the studies published since 2015 (*n* = 23). The existing reviews and descriptive studies (*n* = 7) were limited in scope addressing PSP families in the police sector [41,49,50,67,78], with one review focused on the families of paramedics [39] and a book chapter exploring the impacts of the 9/11 WTC attacks on firefighter families [61]. Certain family members and PSP sectors were underrepresented. Few studies included children in their samples, with an overlap in studies using datasets from the “Children of First Responder and WTC Evacuee Study” [53,54,58,64]. Parents’ perceptions of their children’s experiences provide insight for further study but limit our understanding of the perspectives of the children in PSP families. One children’s study was done in Northern Ireland [41], where both police and their families face significant terrorist threats, which may have limited application in a North American context. With respect to the PSP sector, the families of police were overrepresented (*n =* 28) in the literature, with few references to the families of correctional officers and dispatchers. Studies primarily focused on heterosexual married couples, with most of the non-PSP spouses being female, indicating that there is much to be learned about different family arrangements. Overall, PSP families are understudied in the existing literature, and the strength of this narrative review is that it identifies important gaps where further study is needed.

## 5. Conclusions

The primary objective of this narrative review was to synthesize and explore existing research to identify the unique aspects of PSP family life and provide insights into the relationship between work demands and family processes. Attention was focused on cumulative and acute stressors resulting in disequilibrium or crises for PSP families. It was apparent that the interdependence of family members and the availability of social support are factors that enhance family capabilities, and many PSP families successfully manage the demands by adjusting routines and making accommodations. However, as Cramm et al. [16] note, outcomes depend on the “type, frequency, length, and accumulation of stressors and limited opportunity for a reprieve from stressors, be compressed, family resiliency can be undermined as each of these factors, along with individual family members’ tolerance to withstand them” (p. 630). There is, therefore, a need for PS organizations and communities to be cognizant of the variability and vulnerability of PSP families and to supplement intrafamilial resources with formal and informal supports to enhance their capacity for resilience.

Information, education, and support networks can play a role in the awareness and prevention of mental health issues and help PSP families develop skills to endure cumulative demands. Currently, programs that directly target PSP family resiliency are not widely available, with some evidence-based support extended by the military community (https://woundedwarriors.ca (accessed on 15 August 2021), https://focusproject.org/ (accessed on 15 August 2021)), which can be instructive for developing resiliency programs for PSP families. However, a more comprehensive body of research is needed to inform intervention strategies. Research that is representative of all sectors of PSP families and focuses on the relational aspect of resilience will advance an appreciation of the demands and capabilities within the context of PSP families.

## Figures and Tables

**Table 1:** Summary of articles included in the narrative review.

Author (Year)	Research Design	Sample/Subject (Country)	Summary of Stressors	Key Themes
Agocs et al. (2015) [35]	qualitative	16 female police officers (Canada)	Police work influenced officers’ parenting in a gender-specific context. Nonstandard work and childcare and danger-protection parenting were key themes.	behavior-based conflict, childcare, inequities, nonstandard schedules
Alexander & Walker (1996) [36]	quantitative	409 spouses/partners of police officers (UK)	The impact of the police job on the social life of partners due to shiftwork and long hours was a principal finding. Avoidance, scapegoating, taking work home, and alcohol consumption were means of stress management for police officers. Job-related dangers did not have a significant negative impact on family life.	isolation, nonstandard schedules, stress spill-over, behavior-based conflict
Alrutz et al. (2020) [37]	mixed methods	646 spouses/partners of first responders (New Zealand)	A lack of information and emotional support related to first responder jobs were identified as risk factors for secondary trauma. A lack of organizational support in terms of recognition, communication, and social support for partners was a factor.	identity/culture, isolation, recognition, secondary trauma, social support
Amendola et al. (2021) [38]	quantitative	2 focus groups (8–10) of spouses/partners of police officers and 14 experts (USA)	The findings contributed to an understanding of the work-family conflict. In addition to strain, time, and behavior-based job stressors, the dimensions of emotion, culture, and absorption (work commitment) were added.	public perceptions, stress spillover
Anderson (2019) [39]	review	22 studies paramedics and families (UK)	Childcare conflicts and the impact on the social life of the family were key issues related to shiftwork. Significant emotional demands on the partners of paramedics put them at risk for secondary trauma. Organizational culture was identified as a stressor for workers with ripple effects on families.	nonstandard schedules, stress spillover
Beehr et al. (1995) [40]	quantitative	177 police officers and partners-couples (USA)	Problem-focused, emotion-focused, rugged individualism, and religiosity as coping methods were probed. Religiosity for partners was correlated (negatively) with police officer alcohol consumption and stress levels. Rugged individualism was a coping factor for police officers but not their partners.	coping behaviors, stress spillover
Black (2004) [41]	descriptive	police children (UK)	The author explored the mental health needs of children of police officers and the effects of service-related trauma on family life.	ambiguous loss, children’s mental health, secondary trauma
Bochantin (2010) [11]	qualitative (Ph.D. Thesis)	95 first responders and family members–families (USA)	Processes of meaning-making (sensemaking) as coping strategies were studied in relation to the effects of nonstandard work and emotional labor on family life. The author pointed to humor, risk assessment, and emotion management as factors influencing coping.	behavior-based conflict, communication, coping behaviors, identity/culture, nonstandard schedules
Bochantin (2016) [42]	qualitative	95 first responders and family members–families (USA)	Metaphors used by PSP to describe work-family life were examined. Findings showed that metaphors related to competition, nature/preservation, and ambiguity were frequent, and those associated with balance were uncommon.	communication, meaning-making, compartmentalization, coping behaviors, nonstandard schedules
Bochantin (2017) [43]	qualitative	95 first responders and family members–families (USA)	The distinction between “sensemaking” and “sensetaking” (meaning not shared) was made and the benefits of humor were explored. It was interpreted that humor supported meaningful communication between PSP and their families by easing stress and tension.	communication, coping behaviors, meaning-making
Brimhall et al. (2018) [44]	quantitative	54 male police officers and their spouses/partners–couples (USA)	A secure attachment bond developed through meaningful interactions (responsive and engaged communication) was shown to be a strong indicator of relationship satisfaction. Secure bonds established “goodwill”, which reduced conflict and patterns of withdrawal.	communication, withdrawal, compartmentalization
Brodie & Eppler (2012) [45]	qualitative	7 police officers and their partners–couples (USA)	In addition to the demands of shiftwork, trauma exposure, organizational pressures, and job and financial insecurity were identified as key stressors. Withdrawal, protectiveness, and misunderstanding were reported as challenges for partner communication.	communication, coping behaviors, stress spillover, organizational pressures, financial insecurity, withdrawal, overprotection
Camaro et al. (2020) [46]	quantitative	498 family members and close friends of 911 dispatchers–families (USA)	The perceptions of family and friends regarding the behaviors of 911 dispatchers at home were elicited. Observations of mood changes and withdrawal caused anxiety for family members.	stress spillover
Carrico (2012) [47]	qualitative (Ph.D. Thesis)	5 male firefighters and their partners and children–families (USA)	Findings were analyzed within the framework of family belief systems, organizational patterns, and communication processes. Firefighter job-related stress was reported to impact both workers and families, with the contributions of family members largely unnoticed and unsupported.	coping behaviors, identity/culture, nonstandard schedules, stress spillover, recognition, meaning-making
Carrington (2006) [48]	qualitative (Ph.D. Thesis)	9 police officers (RCMP) and 9 spouses/partners–couples (Canada)	The impacts of shiftwork, postings, and work-related dangers on the marital relationships of RCMP officers were examined. The roles of partners as “half of the badge” focused on the supportive role of partners and a lack of recognition.	communication, identity/culture, inequities, isolation, nonstandard schedules, recognition, social support
Cheema (2016) [49]	descriptive	police families (USA)	Primarily focused on officer-involved domestic violence legislation in the USA, the author shed light on the vulnerability of police officer families and the barriers to accessing support.	behavior-based conflict, stress spillover
Cowlishaw et al. (2008) [50]	review	families of volunteer firefighters (Australia)	The limited research on the role of families in volunteer firefighting revealed that the demands, sacrifices, and lack of recognition have a cumulative impact. The current policies to support families of volunteer firefighters were deemed inadequate or nonexistent.	identity/culture, nonstandard schedules, stress spillover
Cowlishaw et al. (2010) [51]	quantitative	102 first responders and their spouses/partners–couples (Australia)	The stress associated with volunteer emergency work impacted partners in the sample. Withdrawn behavior of volunteers resulted in distress for partners and diminished the quality of relationships.	ambiguous loss, crossover, stress spillover
Davidson et al. (2006) [52]	quantitative	103 police officers and their spouses/partners–couples (Australia)	Findings showed that partners of traumatized police officers also experienced symptoms. Avoidance and emotional numbing symptoms in police officers that can manifest in poorer communication were related to significant distress for partners.	crossover, secondary trauma
Duarte et al. (2006) [53]	quantitative	8236 New York school children–first responders (USA)	Probable PTSD among children of first responders who responded to the 9/11 WTC attacks was asserted based on symptomatology. Higher rates of mental health problems were found in emergency medical technicians’ children when compared with the children of firefighters.	children’s mental health, identity/culture, secondary trauma
Duarte et al. (2019) [54]	quantitative	556 children of first responders involved with the 9/11 WTC attacks (USA)	Children’s knowledge of first responder parents’ involvement in the 9/11 WTC attacks was proposed as a factor in mental health outcomes. The study revealed that many children had little awareness of parental involvement.	children’s mental health, communication
Duxbury & Higgins (2012) [55]	quantitative	4500 police officers (Canada)	Findings suggested that police organizations have not adjusted to changes in family life, particularly the predominance of dual-earner households. A lack of control over the work environment, work overload, high family demands, and organizational culture were identified as factors that interfere with the satisfaction of both work and family roles.	organizational pressures, childcare, role overload
Duxbury et al. (2021) [56]	quantitative	616 male and 264 female police officers (Canada)	Although findings indicated that police officers were progressing to close the gender gap in families, organizations and organizational culture were found to oppose this shift. The stigmatization of supports intended to help police officers balance work and family life were shown to interfere with uptake.	inequities, organizational pressures
Ewles (2019) [57]	mixed methods (Ph.D. Thesis)	179 police officers and 38 spouses/partners–couples (Canada)	Family interactions were influenced by work pressures, family demands (e.g., childcare), and financial concerns. Police officer behaviors, including withdrawal, irritability, and maladaptive coping (alcohol consumption), affected the quality of family relationships.	ambiguous loss, coping behaviors, identity/culture, nonstandard schedules, social support
Friese (2020) [6]	mixed methods	171 spouses/partners of police officers (USA)	Most participants reported significant stress related to their partner’s police work. Maladaptive coping strategies (e.g., alcohol consumption, withdrawal) were prevalent. Sleep issues, secondary trauma, and disruptions to plans were revealed in focus groups. Common positive coping skills included self-care and exercise.	secondary trauma, crossover, isolation, resilience, nonstandard work, secondary trauma
Helfers et al. (2021) [21]	qualitative	19 children of police officers (USA)	Feeling protected and knowledgeable about the law were reported as benefits of having a police parent. Children also felt restricted, overprotected, and experienced stress. They worried about the police parents’ safety on the job and negative public perceptions.	behavior-based conflict, children’s mental health, public perceptions, social support
Hill et al. (2020) [22]	qualitative	9 partners and 1 sibling of firefighters–families (UK)	Demands, sacrifices, flexibility, and the importance of social support from other firefighter families were highlighted. The consequences for family members who support firefighters included stress spillover and crossover.	coping behaviors, crossover, identity/culture, isolation, nonstandard schedules, recognition, stress spillover
Hoven et al. (2009) [58]	quantitative	350 Israeli and 900 New York first responder children (USA)	A proposed study on the effects of parental exposure to mass violence (9/11 WTC attacks) on the mental health of their children theorized that the effects are influenced by the child’s understanding and perception of the event and the parent’s role.	children’s mental health, crossover, identity/culture, secondary trauma
Jackson & Maslach (1982) [59]	qualitative	142 male police officers and their spouses/partners–couples (USA)	Job stress (measured) was shown to be related to police officers exhibiting irritability and anger and disengaging from family life. The involvement of the police officer in family life was associated with relationship satisfaction. The authors point to organizations to help families manage job-related stress.	communication, coping behaviors, couple relationships, isolation, social support, stress spillover
Johnson et al. (2005) [60]	quantitative	413 police officers (USA)	Forty percent of a random sample of police officers reported behaving violently towards their partners. Burnout, authoritarian spillover, and alcohol consumption were shown to be mediators for the indirect effects of exposure to violence on spousal violence.	behavior-based conflict, risk factors, organizational processes
Karaffa et al. (2015) [7]	quantitative	82 police officers and 89 spouses/partners–couples (USA)	A needs assessment related to the effect of police work on marital relationships identified financial concerns, nonstandard work hours, public perceptions, and negative behaviors as stressors. Resources included spirituality, open communication, pride, and support from extended family, friends, and other police families.	behavior-based conflict, identity/culture, nonstandard schedules, public perceptions, social support, stress spillover
Kelly (2012) [61]	descriptive (book chapter)	firefighters and their families (USA)	The impacts of firefighters’ behavior on their families in the aftermath of the 9/11 WTC attacks were examined. The association between family experiences of unpredictability, insecurity, and ambiguous loss have and firefighters’ PTSD symptoms of avoidance and arousal were highlighted.	ambiguous loss, stress spillover
King & DeLongis (2014) [62]	quantitative	87 paramedics and their spouses/partners–couples (Canada)	Rumination and withdrawal were maladaptive coping behaviors found in both partners and paramedics. Job-related stress reduced the quality of interactions and maladaptive coping escalated tensions.	coping behaviors, withdrawal, crossover
King (2013) [63]	mixed methods (Ph.D. Thesis)	87 paramedics and their spouses/partners–couples (Canada)	Findings associated with the transmission of job-related stress to partners revealed that paramedics sampled were at high risk for depression, PTSD, and burnout. Significant crossover of negative affect to partners was also evident. Compensatory behaviors aimed at avoiding conflict often increase tensions between partners.	coping behaviors, crossover, stress spillover
Kishon et al. (2020) [64]	quantitative	208 children of Israeli first responders (USA)	Evidence supported an association between mental health outcomes for children and first responder parents’ trauma exposure and a further risk of secondary trauma in younger children who are more dependent on the trauma-exposed parent.	children’s mental health, secondary trauma
Lambert et al. (2004) [14]	quantitative	272 correctional staff (USA)	Organizational pressures associated with a lack of control over procedures and scheduling were factors in work-family conflicts. Time-based stressors (shiftwork) were more significant for younger versus older correctional officers. Organizational inflexibility was identified as a challenge.	nonstandard schedules, organizational pressures
Landers et al. (2020) [8]	qualitative	8 spouses/partners of police officers (USA)	The effect of police officers’ responses to traumatic events (reactivity, isolation) on partners and the direct impact of the event on partners (fear, hypervigilance) were reported. Increased couple communication, mutual support, informal social support, routines, reframing, and religiosity were disclosed as coping strategies.	coping behaviors, secondary trauma, communication, meaning-making
Maynard et al. (1980) [65]	qualitative	42 female spouses/partners of police officers (USA)	Drawing on resilience research, coping patterns were analyzed in terms of family functioning. Self-reliance, accepting the demands of the job, social support, and role maintenance were strategies that partners used to maintain organization and stability and manage stress.	coping behaviors, social support
Meffert et al. (2014) [66]	quantitative	71 police officers and their spouses/partners–couples (USA)	Findings showed that partners’ perceptions of police officers’ PTSD symptoms were linked to their distress and put them at risk for secondary trauma and relationship violence.	crossover, secondary trauma, meaning-making
Miller (2007) [67]	descriptive	police families (USA)	The article pointed to divided loyalties (e.g., overwork), overprotectiveness, compartmentalization, and hypervigilance on the part of police officers that impacted family relationships. Shared identities and the cumulative stress load were underscored.	behavior-based conflict, compartmentalization, coping behaviors, identity/culture, stress spillover
Pfefferbaum et al. (2002) [68]	mixed methods	27 spouses/partners of firefighters (USA)	Findings suggest that partners of firefighters involved with the Oklahoma City bombing (1995) coped well in the aftermath, with few exhibiting PTSD symptoms. Both positive and negative changes in couple relationships were reported. Social support from family and friends was the primary mechanism for coping.	secondary trauma, social support
Porter & Henricksen (2016) [9]	qualitative	6 spouses/partners of first responders (USA)	Safety, stress, pride, civic-mindedness, identity, and finances were themes revealed. Both the stressors and benefits of this way of life for first responder families were reported.	ambiguous loss, behavior-based conflict, identity/culture, nonstandard schedules
Regehr (2005) [69]	qualitative	14 spouses/partners of paramedics (Canada)	Findings showed the effects of paramedic trauma exposure on families were intensified by the unpredictability of shiftwork and overtime. Cumulative demands were associated with behavior-based conflict, withdrawal, and the risk of secondary trauma. A lack of organizational support for families was identified.	communication, isolation, nonstandard schedules, secondary trauma, stress spillover
Regehr et al. (2005) [10]	qualitative	14 spouses/partners of firefighters (Canada)	Nonstandard work, trauma exposure, and organizational culture were the key areas of inquiry. Poor communication, ambiguous loss, isolation, managing spillover, and a lack of recognition and organizational support for families were challenges. Pride in the role of the firefighter was noted as a benefit for families.	ambiguous loss, childcare, crossover, identity/culture, nonstandard schedules, social support, stress spillover
Roberts & Levenson (2001) [70]	mixed methods	19 male police officers and their spouses/partners–couples (USA)	Job stress was shown to have a more significant impact on police officers’ relationships with partners than physical exhaustion. Job stress was found to increase negative affect and emotional distance in both partners during interactions.	crossover, nonstandard schedules
Roberts et al. (2013) [71]	mixed methods	17 male police officers and their spouses/partners–couples (USA)	Spillover effects of job stress had variable impacts on marital relationships. Avoidance was related to marital dissatisfaction, whereas attending to negative emotions supported marital satisfaction for partners.	coping behaviors, spillover
Roth & Moore (2009) [72]	qualitative	11 spouses/partners and 1 parent of first responders–families (USA)	Negotiation of roles, open communication, having independent interests, allowing the first responders alone time, supporting the first responders emotionally, and concerns for the first responders’ safety were themes related to coping strategies that emerged. Shiftwork and overtime were identified as disruptive but manageable.	childcare, communication, identity/culture, nonstandard schedules, resilience, stress spillover
Sommerfeld et al. (2017) [73]	qualitative	10 firefighters and 9 of their spouses/partners–couples (Canada)	Partners identified shift work, “the brotherhood”, trauma exposure, and health and safety as factors influencing job-related stress. Flexibility, extended family and friends, and positive public perceptions were highlighted as supports for the worker. The researchers concluded that interventions should account for the effects of job-related stress on both firefighters and their spouses.	childcare, coping behaviors, identity/culture, isolation, nonstandard schedules, public perceptions
Thompson (2012) [74]	qualitative (Master’s Thesis)	8 police officer spouses/partners (female) (Canada)	Lifestyle, social support, humor, vicarious trauma, communication, and the importance of the partner role were themes related to the effect of job-related stress and coping on marital relationships.	coping behaviors, isolation, nonstandard schedules, recognition, secondary trauma, stress spillover, social support
Thompson et al. (2001) [75]	qualitative (book chapter)	29 female police officers (USA)	Operational stress, organizational culture, and management were identified as sources of stress for workers. The importance of social support at work and home was prominent. Spillover of negative moods was reported to impact family relationships.	stress spillover
Tuttle et al. (2018) [12]	quantitative	1180 married police officers (USA)	Job-related stress affected communication and emotion regulation, negatively impacting marital satisfaction. Organizational pressures and emotional stress spillover had a significant negative impact on marital relationships.	communication, nonstandard schedules, stress spillover
Waddell et al. (2020) [76]	qualitative	22 spouses/partners of first responders and veterans (Australia)	Partners reported that invisibility and a lack of support diminished their ability to support first responders and veterans with PTSD. As partners adapted to new roles and responsibilities within the family, extrafamilial factors were identified as barriers.	recognition, secondary trauma, organizational processes
Watkins et al. (2021) [77]	qualitative	10 focus groups of firefighters and spouses/partners–couples (USA)	Shiftwork and sleep loss impacted family relationships, including poor communication and emotional unavailability. The prioritization of family time and sleep support from families were key themes.	ambiguous loss, couple relationships, nonstandard schedules
Woody (2006) [78]	descriptive	police and their families (USA)	Organizational pressures, dangers, and public disdain for police officers were described as factors contributing to high-stress levels for workers. These factors were associated with behavior-based conflict and stress spillover for families.	public perceptions, stress spillover

**Table 2:** Summary of findings.

Context	Issues	Findings
Structural Interference	Role overload Family time Routines Ambivalence	Family life cycle influences family demands. Shiftwork that allows couples to divide childcare responsibilities can reduce couple time. There is a lack of institutional childcare for nonstandard work hours. Relocation is disruptive to non-PSP partners’ careers and social support. Sleep deprivation and disrupted sleep affect the quantity and quality of family interactions. PSP who work holidays, weekends, and evenings miss family and community events. Unpredictability of work disrupts plans for family time. Shiftwork and call-ins interfere with routines such as mealtimes and bedtimes. Division of labor within the home is often gendered and inequitable.
Emotional Interference	Behavior-based conflict Ambiguous loss Crossover Identity	Hypervigilance; authoritarian behaviors of PSP spillover into family roles (e.g., overprotective parenting). Withdrawal causes a breakdown in family communication. Young children who often do not understand work demands experience sadness and anger. Both non-disclosure and superfluous detail about an event can cause distress for families. PSP stress can have a ripple effect in families. PTSD symptoms experienced by PSP can impact the mental health of family members. Public perceptions of PSP, both positive and negative, affect their families. PSP families are a source of support and information for other PSP families. The significant role of families in the PSP career is inadequately recognized and supported.
Risk of Injury or Death	Life-threatening work Injuries	Both partners and children worry about PSPs’ safety. Commentary and events shared on social media increase distress for PSP families. Information about safety procedures alleviates fears. PSP families early in their careers often have more fears. Partners are typically the primary caregivers when PSP experience physical or operational stress injuries. PSP families have financial concerns related to the risk of injury or death.

## Data Availability

Not applicable.

## References

[1] Oliphant R. (2016). Healthy Minds, Safe Communities, Supporting Our Public Safety Officers through a National Strategy for Operational Stress Injuries: Report of the Standing Committee on Public Safety and National Security.

[2] Anderson G.S., Di Nota P.M., Groll D., Carleton R.N. (2020). Peer Support and Crisis-Focused Psychological Interventions Designed to Mitigate Post-Traumatic Stress Injuries among Public Safety and Frontline Healthcare Personnel: A Systematic Review. Int. J. Environ. Res. Public Health.

[3] Carleton R.N., Afifi T.O., Taillieu T., Turner S., Krakauer R., Anderson G.S., MacPhee R.S., Ricciardelli R., Cramm H.A., Groll D. (2019). Exposures to Potentially Traumatic Events among Public Safety Personnel in Canada. Can. J. Behav. Sci..

[4] Krakauer R.L., Stelnicki A.M., Carleton R.N. (2020). Examining Mental Health Knowledge, Stigma, and Service Use Intentions among Public Safety Personnel. Front. Psychol..

[5] Ricciardelli R., Czarnuch S., Afifi T.O., Taillieu T., Carleton R.N. (2020). Public Safety Personnel’s Interpretations of Potentially Traumatic Events. Occup. Med..

[6] Friese K.M. (2020). Cuffed Together: A Study on How Law Enforcement Work Impacts the Officer’s Spouse. Int. J. Police Sci. Manag..

[7] Karaffa K., Openshaw L., Koch J., Clark H., Harr C., Stewart C. (2015). Perceived Impact of Police Work on Marital Relationships. Fam. J..

[8] Landers A.L., Dimitropoulos G., Mendenhall T.J., Kennedy A., Zemanek L. (2020). Backing the Blue: Trauma in Law Enforcement Spouses and Couples. Fam. Relat..

[9] Porter K.L., Henriksen R.C. (2016). The Phenomenological Experience of First Responder Spouses. Fam. J..

[10] Regehr C., Dimitropoulos G., Bright E., George S., Henderson J. (2005). Behind the Brotherhood: Rewards and Challenges for Wives of Firefighters. Fam. Relat..

[11] Bochantin J.E. (2010). Sensemaking in a High-Risk Lifestyle: The Relationship between Work and Family for Public Safety Families. Ph.D. Dissertation.

[12] Tuttle B.M., Giano Z., Merten M.J. (2018). Stress Spillover in Policing and Negative Relationship Functioning for Law Enforcement Marriages. Fam. J..

[13] O’Neill O.A., Rothbard N.P. (2017). Is Love All You Need? The Effects of Emotional Culture, Suppression, and Work–Family Conflict on Firefighter Risk-Taking and Health. Acad. Manag. J..

[14] Lambert E.G., Hogan N.L., Barton S.M. (2004). The Nature of Work-Family Conflict among Correctional Staff: An Exploratory Examination. Crim. Justice Rev..

[15] Walsh F. (2003). Family Resilience: A Framework for Clinical Practice. Fam. Process.

[16] Cramm H., Norris D., Venedam S., Tam-Seto L. (2018). Toward a Model of Military Family Resiliency: A Narrative Review: Military Family Resiliency. J. Fam. Theory Rev..

[17] Ricciardelli R., Carleton R.N., Groll D., Cramm H. (2018). Qualitatively Unpacking Canadian Public Safety Personnel Experiences of Trauma and Their Well-Being. Can. J. Criminol. Crim. Justice.

[18] Daigle P. (2013). On the Homefront: Assessing the Wellbeing of Canada’s Military Families in the New Millennium.

[19] Smith C.R. (2020). Annual Report 2019–2020.

[20] Jeklin A.T., Davies H.W., Bredin S.S.D., Perrotta A.S., Hives B.A., Meanwell L., Warburton D.E.R. (2021). Using a Biomathematical Model to Assess Fatigue Risk and Scheduling Characteristics in Canadian Wildland Firefighters. Int. J. Wildland Fire.

[21] Helfers R.C., Reynolds P.D., Scott D.M. (2021). Being a Blue Blood: A Phenomenological Study on the Lived Experiences of Police Officers’ Children. Police Q..

[22] Hill R., Sundin E., Winder B. (2020). Work–Family Enrichment of Firefighters: “Satellite Family Members”, Risk, Trauma and Family Functioning. Int. J. Emerg. Serv..

[23] Carleton R.N., Afifi T.O., Turner S., Taillieu T., Duranceau S., LeBouthillier D.M., Sareen J., Ricciardelli R., MacPhee R.S., Groll D. (2017). Mental Disorder Symptoms among Public Safety Personnel in Canada. Can J. Psychiatry.

[24] Boss P. (1992). Primacy of Perception in Family Stress Theory and Measurement. J. Fam. Psychol..

[25] Masten A.S. (2013). Competence, Risk, and Resilience in Military Families: Conceptual Commentary. Clin. Child Fam. Psychol. Rev..

[26] Meadows S.O., Beckett M.K., Bowling K., Golinelli D., Fisher M.P., Martin L.T., Meredith L.S., Osilla K.C. (2016). Family Resilience in the Military: Definitions, Models, and Policies. Rand Health Q..

[27] Saltzman W.R., Lester P., Beardslee W.R., Layne C.M., Woodward K., Nash W.P. (2011). Mechanisms of Risk and Resilience in Military Families: Theoretical and Empirical Basis of a Family-Focused Resilience Enhancement Program. Clin. Child Fam. Psychol. Rev..

[28] Mays N., Pope C., Popay J. (2005). Systematically Reviewing Qualitative and Quantitative Evidence to Inform Management and Policy-Making in the Health Field. J. Health Serv. Res. Policy.

[29] Green B.N., Johnson C.D., Adams A. (2006). Writing Narrative Literature Reviews for Peer-Reviewed Journals: Secrets of the Trade. J. Chiropr. Med..

[30] Ferrari R. (2015). Writing Narrative Style Literature Reviews. Med. Writ..

[31] Collins J.A., Fauser B.C.J.M. (2005). Balancing the Strengths of Systematic and Narrative Reviews. Hum. Reprod. Update.

[32] Braun V., Clarke V. (2006). Using Thematic Analysis in Psychology. Qual. Res. in Psych.

[33] Clarke V., Braun V. (2013). Teaching Thematic Analysis. Psychologist.

[34] Page M.J., McKenzie J.E., Bossuyt P.M., Boutron I., Hoffmann T.C., Mulrow C.D., Shamseer L., Tetzlaff J.M., Akl E.A., Brennan S.E. (2021). The PRISMA 2020 Statement: An Updated Guideline for Reporting Systematic Reviews. J. Clin. Epidemiol..

[35] Agocs T., Langan D., Sanders C.B. (2015). Police Mothers at Home: Police Work and Danger-Protection Parenting Practices. Gend. Soc..

[36] Alexander D.A., Walker L.G. (1996). The Perceived Impact of Police Work on Police Officers’ Spouses and Families. Stress Med..

[37] Alrutz A.S., Buetow S., Cameron L.D., Huggard P.K. (2020). What Happens at Work Comes Home. Healthcare.

[38] Amendola K.L., Valdovinos Olson M., Grieco J., Robbins T.G. (2021). Development of a Work–Family Conflict Scale for Spouses or Partners of Police Officers. Policing.

[39] Anderson L. (2019). The Impact of Paramedic Shift Work on the Family System: A Literature Review. Br. Paramed. J..

[40] Beehr T.A., Johnson L.B., Nieva R. (1995). Occupational Stress: Coping of Police and Their Spouses. J. Organ. Behav..

[41] Black A. (2004). The Treatment of Psychological Problems Experienced by the Children of Police Officers in Northern Ireland. Child Care Pract..

[42] Bochantin J.E. (2016). “Morning Fog, Spider Webs, and Escaping from Alcatraz”: Examining Metaphors Used by Public Safety Employees and Their Families to Help Understand the Relationship between Work and Family. Commun. Monogr..

[43] Bochantin J.E. (2017). “Ambulance Thieves, Clowns, and Naked Grandfathers”: How PSEs and Their Families Use Humorous Communication as a Sensemaking Device. Manag. Commun. Q..

[44] Brimhall A.S., Bonner H.S., Tyndall L., Jensen J.F. (2018). ARE You There for Me? The Relationship between Attachment, Communication, and Relationship Satisfaction of Law Enforcement Officers and Their Partners. J. Couple Relatsh. Ther..

[45] Brodie P.J., Eppler C. (2012). Exploration of Perceived Stressors, Communication, and Resilience in Law-Enforcement Couples. J. Fam. Psychother..

[46] Camaro A., Belmonte E., Demar J., Timm A. (2020). The Impact of 911 Telecommunications on Family and Social Interaction. Ann. Emerg. Dispatch Response.

[47] Carrico C. (2012). A Look inside Firefighter Families: A Qualitative Study. Ph.D. Dissertation.

[48] Carrington J.L. (2006). Elements of and Strategies for Maintaining a Police Marriage: The Lived Perspectives of Royal Canadian Mounted Police Officers and Their Spouses. Ph.D. Dissertation.

[49] Cheema R. (2016). Black and Blue Bloods: Protecting Police Officer Families from Domestic Violence: Black and Blue Blood. Fam. Court Rev..

[50] Cowlishaw S., Evans L., McLennan J. (2008). Families of Rural Volunteer Firefighters. Rural Soc..

[51] Cowlishaw S., Evans L., McLennan J. (2010). Work–Family Conflict and Crossover in Volunteer Emergency Service Workers. Work Stress.

[52] Davidson A.C., Berah E., Moss S. (2006). The Relationship between the Adjustment of Australian Police Officers and Their Partners. Psychiatr. Psychol. Law.

[53] Duarte C.S., Hoven C.W., Wu P., Bin F., Cotel S., Mandell D.J., Nagasawa M., Balaban V., Wernikoff L., Markenson D. (2006). Posttraumatic Stress in Children with First Responders in Their Families. J. Trauma. Stress.

[54] Duarte C.S., Eisenberg R., Musa G.J., Addolorato A., Shen S., Hoven C.W. (2019). Children’s Knowledge about Parental Exposure to Trauma. J. Child Adolesc. Trauma.

[55] Duxbury L., Higgins C. Caring for and About Those Who Serve: Work-Life Conflict and Employee Well Being within Canada’s Police Departments. https://sprott.carleton.ca/wp-content/uploads/Duxbury-Higgins-Police2012_fullreport.pdf.

[56] Duxbury L., Bardoel A., Halinski M. (2021). ‘Bringing the Badge Home’: Exploring the Relationship between Role Overload, Work-Family Conflict, and Stress in Police Officers. Polic. Soc..

[57] Ewles G. (2019). Enhancing Organizational Support for Emergency First Responders and Their Families: Examining the Role of Personal Support Networks after the Experience of Work-Related Trauma. Ph.D. Thesis.

[58] Hoven C.W., Duarte C.S., Wu P., Doan T., Singh N., Mandell D.J., Bin F., Teichman Y., Teichman M., Wicks J. (2009). Parental Exposure to Mass Violence and Child Mental Health: The First Responder and WTC Evacuee Study. Clin. Child Fam. Psychol. Rev..

[59] Jackson S.E., Maslach C. (1982). After-Effects of Job-Related Stress: Families as Victims. J. Organ. Behav..

[60] Johnson L.B., Todd M., Subramanian G. (2005). Violence in Police Families: Work-Family Spillover. J. Fam. Violence.

[61] Kelly K.V. (2018). Heroes at Home: The Transmission of Trauma in Firefighters’ Families. Lost in Transmission.

[62] King D.B., DeLongis A. (2014). When Couples Disconnect: Rumination and Withdrawal as Maladaptive Responses to Everyday Stress. J. Fam. Psychol..

[63] King D.B. (2013). Daily Dynamics of Stress in Canadian Paramedics and Their Spouses. Ph.D. Dissertation.

[64] Kishon R., Geronazzo-Alman L., Teichman M., Teichman Y., Cheslack-Postava K., Fan B., Duarte C.S., Wicks J., Musa G.J., Djalovski A. (2020). Parental Occupational Exposure Is Associated with Their Children’s Psychopathology: A Study of Families of Israeli First Responders. J. Occup. Environ. Med..

[65] Maynard P., Maynard N., McCubbin H.I., Shao D. (1980). Family Life and the Police Profession: Coping Patterns Wives Employ in Managing Job Stress and the Family Environment. Fam. Relat..

[66] Meffert S.M., Henn-Haase C., Metzler T.J., Qian M., Best S., Hirschfeld A., McCaslin S., Inslicht S., Neylan T.C., Marmar C.R. (2014). Prospective Study of Police Officer Spouse/Partners: A New Pathway to Secondary Trauma and Relationship Violence?. PLoS ONE.

[67] Miller L. (2007). Police Families: Stresses, Syndromes, and Solutions. Am. J. Fam. Ther..

[68] Pfefferbaum B., North C.S., Bunch K., Wilson T.G., Tucker P., Schorr J.K. (2002). The Impact of the 1995 Oklahoma City Bombing on the Partners of Firefighters. J. Urban Health.

[69] Regehr C. (2005). Bringing the Trauma Home: Spouses of Paramedics. J. Loss Trauma.

[70] Roberts N.A., Levenson R.W. (2001). The Remains of the Workday: Impact of Job Stress and Exhaustion on Marital Interaction in Police Couples. J. Marriage Fam..

[71] Roberts N.A., Leonard R.C., Butler E.A., Levenson R.W., Kanter J.W. (2013). Job Stress and Dyadic Synchrony in Police Marriages: A Preliminary Investigation. Fam. Process.

[72] Roth S.G., Moore C.D. (2009). Work-Family Fit: The Impact of Emergency Medical Services Work on the Family System. Prehosp. Emerg. Care.

[73] Sommerfeld A., Wagner S.L., Harder H.G., Schmidt G. (2017). Behavioral Health and Firefighters: An Intervention and Interviews with Canadian Firefighters. J. Loss Trauma.

[74] Thompson A.J. (2012). Operational Stress and the Police Marriage: A Narrative Study of Police Spouses. Master’s Dissertation.

[75] Thompson B., Kirk-Brown A., Brown D., Hancock P.A., Desmond P.A. (2001). Women Police: The Impact of Work Stress on Family Members. Stress, Workload, and Fatigue.

[76] Waddell E., Lawn S., Roberts L., Henderson J., Venning A., Redpath P. (2020). “Why Do You Stay?”: The Lived-experience of Partners of Australian Veterans and First Responders with Posttraumatic Stress Disorder. Health Soc. Care Community.

[77] Watkins S.L., Shannon M.A., Hurtado D.A., Shea S.A., Bowles N.P. (2021). Interactions between Home, Work, and Sleep among Firefighters. Am. J. Ind. Med..

[78] Woody R.H. (2006). Family Interventions with Law Enforcement Officers. Am. J. Fam. Ther..

[79] Jackson S.E., Zedeck S., Summers E. (1985). Family Life Disruptions: Effects of Job-Induced Structural and Emotional Interference. Acad. Manag. J..

[80] Strazdins L., Clements M.S., Korda R.J., Broom D.H., D’Souza R.M. (2006). Unsociable Work? Nonstandard Work Schedules, Family Relationships, and Children’s Well-Being. J. Marriage Fam..

[81] Offer S. (2013). Assessing the Relationship between Family Mealtime Communication and Adolescent Emotional Well-Being Using the Experience Sampling Method. J. Adolesc..

[82] Perry-Jenkins M., Goldberg A.E., Pierce C.P., Sayer A.G. (2007). Shift Work, Role Overload, and the Transition to Parenthood. J. Marriage Fam..

[83] Rapoport B., Le Bourdais C. (2008). Parental Time and Working Schedules. J. Popul. Econ..

[84] Strazdins L., Korda R.J., Lim L.L.-Y., Broom D.H., D’Souza R.M. (2004). Around-the-Clock: Parent Work Schedules and Children’s Well-Being in a 24-h Economy. Soc. Sci. Med..

[85] Wight V.R., Raley S.B., Bianchi S.M. (2008). Time for Children, One’s Spouse and Oneself among Parents Who Work Nonstandard Hours. Soc. Forces.

[86] Greenhaus J.H., Beutell N.J. (1985). Sources of Conflict between Work and Family Roles. Acad. Manag. Rev..

[87] Bakker A.B., Demerouti E., Dollard M.F. (2008). How Job Demands Affect Partners’ Experience of Exhaustion: Integrating Work-Family Conflict and Crossover Theory. J. Appl. Psychol..

[88] Norris D., Cramm H., Eichler M., Tam-Seto L., Smith-Evans K. The Impact of Operational Stress Injuries on Canadian Armed Forces Military and Veteran Families. https://cimvhr.ca/documents/Appendix%20B.pdf?cimlang=en.

[89] Rutter M. (2012). Resilience as a Dynamic Concept. Dev. Psychopathol..

[90] Paley B., Lester P., Mogil C. (2013). Family Systems and Ecological Perspectives on the Impact of Deployment on Military Families. Clin. Child Fam. Psychol. Rev..

[91] Henry C.S., Sheffield Morris A., Harrist A.W. (2015). Family Resilience: Moving into the Third Wave: Family Resilience. Fam. Relat..

[92] Crane M.F., Searle B.J. (2016). Building Resilience through Exposure to Stressors: The Effects of Challenges versus Hindrances. J. Occup. Health Psychol..

[93] Patterson J.M. (1988). Families Experiencing Stress: I. The Family Adjustment and Adaptation Response Model: II. Applying the FAAR Model to Health-Related Issues for Intervention and Research. Fam. Syst. Med..

[94] Arnold A.L., Lucier-Greer M., Mancini J.A., Ford J.L., Wickrama K.A.S. (2017). How Family Structures and Processes Interrelate: The Case of Adolescent Mental Health and Academic Success in Military Families. J. Fam. Issues.

[95] Hill R. (1958). Generic Features of Families under Stress. Soc. Casework.

[96] Olson D.H. (2000). Circumplex Model of Marital and Family Systems. J. Fam. Ther..

[97] Mancini J.A., O’Neal C.W., Martin J.A., Bowen G.L. (2018). Community Social Organization and Military Families: Theoretical Perspectives on Transitions, Contexts, and Resilience: Theoretical Perspectives on Military Families. J. Fam. Theory Rev..

[98] Lavee Y., Olson D.H. (1991). Family Types and Response to Stress. J. Marriage Fam..

[99] Olson D.H., Waldvogel L., Schlieff M. (2019). Circumplex Model of Marital and Family Systems: An Update. J. Fam. Theory Rev..

[100] McCubbin H.I., McCubbin M.A. (1988). Typologies of Resilient Families: Emerging Roles of Social Class and Ethnicity. Fam. Relat..

[101] Lucier-Greer M., Arnold A.L., Mancini J.A., Ford J.L., Bryant C.M. (2015). Influences of Cumulative Risk and Protective Factors on the Adjustment of Adolescents in Military Families. Fam. Relat..

[102] Riggs S.A., Riggs D.S. (2011). Risk and Resilience in Military Families Experiencing Deployment: The Role of the Family Attachment Network. J. Fam. Psychol..

[103] Easterbrooks M.A., Ginsburg K., Lerner R.M. (2013). Resilience among Military Youth. Future Child..

[104] Bronfenbrenner U. (1977). Toward an Experimental Ecology of Human Development. Am. Psychol..

